# Modeling Insights into *Haemophilus influenzae* Type b Disease, Transmission, and Vaccine Programs

**DOI:** 10.3201/eid1801.110336

**Published:** 2012-01

**Authors:** Michael L. Jackson, Charles E. Rose, Amanda Cohn, Fatima Coronado, Thomas A. Clark, Jay D. Wenger, Lisa Bulkow, Michael G. Bruce, Nancy E. Messonnier, Thomas W. Hennessy

**Affiliations:** Centers for Disease Control and Prevention, Atlanta, Georgia, USA

**Keywords:** Haemophilus influenzae type b, Hib, vaccines, vaccination, immunization programs, mathematical model, bacteria, disease transmission, United States, England, Wales, Alaska, susceptible populations, herd immunity

## Abstract

In response to the 2007–2009 *Haemophilus influenzae* type b (Hib) vaccine shortage in the United States, we developed a flexible model of Hib transmission and disease for optimizing Hib vaccine programs in diverse populations and situations. The model classifies population members by age, colonization/disease status, and antibody levels, with movement across categories defined by differential equations. We implemented the model for the United States as a whole, England and Wales, and the Alaska Native population. This model accurately simulated Hib incidence in all 3 populations, including the increased incidence in England/Wales beginning in 1999 and the change in Hib incidence in Alaska Natives after switching Hib vaccines in 1996. The model suggests that a vaccine shortage requiring deferral of the booster dose could last 3 years in the United States before loss of herd immunity would result in increasing rates of invasive Hib disease in children <5 years of age.

## Abstract 2

Flexible simulation model use can optimize vaccination programs and response to changes in vaccine supply.

Routine use of *Haemophilus influenzae* type b (Hib) conjugate vaccines has dramatically reduced the incidence of Hib disease in children <5 years of age in numerous populations (*1**–**4*). Vaccination programs have also led to herd immunity through reduced Hib transmission, as shown by declines in the prevalence of oropharyngeal Hib colonization among vaccinated children and unvaccinated children and adults (*2**,**4**–**6*). However, even successful vaccination programs have not eliminated Hib colonization (*7**,**8*). Thus, the continued success of Hib control programs depends on maintaining age-appropriate Hib vaccine coverage. Such coverage can, however, be threatened by changes in vaccine supply, as indicated by the 2007–2009 Hib vaccine shortage in the United States (*9**,**10*).

To manage that shortage, the Centers for Disease Control and Prevention and partner organizations recommended that providers defer giving the 12–15-month booster dose to all children except those at high risk for invasive Hib disease (*9*). This recommendation was based on expert opinion about the predicted effects of a shortage initially expected to last <9 months (*9*). When it became clear that the shortage would last longer, we sought to develop a model of Hib transmission and disease to predict the effects of continued booster dose deferral and to guide vaccine policy. Such a model could also be useful for optimizing the introduction of Hib vaccines into new populations. Furthermore, it could provide insights into the dynamics of Hib transmission and colonization, which would inform the uncertainty over the types of Hib vaccines that are most appropriate for populations at high risk for invasive Hib disease, such as Alaska Natives (*11*). We present the model and show its application to various populations and vaccination scenarios.

## Methods

### Model Structure, Parameters, and Starting Conditions

**Figure 1:**
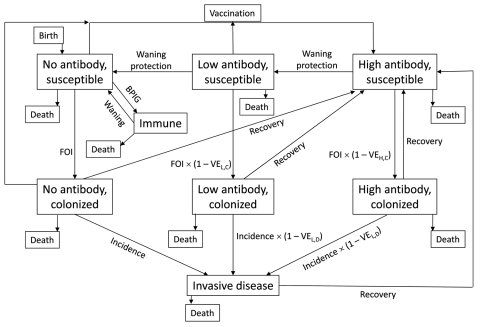
Structure of *Haemophilus influenzae* type b (Hib) simulation model. Persons are born into the no-antibody, disease-susceptible state and can die in any of the model states. Hib-susceptible persons become colonized based on the force of infection (FOI), which is reduced by protection from low (VE_L,C_) or high (VE_H,C_) antibody levels. Persons colonized with Hib develop invasive disease, which is reduced by protection from low (VE_L,D_) or high (VE_H,D_) antibody levels. Colonized and diseased persons recover to the high-antibody, disease-susceptible state. As immunity wanes, susceptible persons move from high to low antibody and from low to no antibody. Some persons are vaccinated and move from no or low antibody states to the high antibody state. For the Alaska Native population, use of bacterial polysaccharide immunoglobulin (BPIG) starting at birth temporarily moves persons to an immune state; as BPIG wanes, immune persons return to the susceptible state. See Technical Appendix 1 for a formal description of the model structure.

We developed an age-structured mathematical model to describe Hib transmission, colonization, and disease (Figure 1). The model assumes that populations can be divided into mutually exclusive states on the basis of age, Hib antibody levels (high, low, and none), and Hib infection status (susceptible, colonized, and diseased), with an additional state (immune) for infants passively immunized with bacterial polysaccharide immunoglobulin. This model can be expressed as a set of partial differential equations (Technical Appendix 1), with rate parameters governing the movement of the population between model states. As an example, the age-specific force of infection (λ(*a*)) is the rate at which susceptible persons of age *a* become colonized. We set values for the rate parameters by using published and unpublished data on birth and death rates, Hib colonization and incidence, and Hib vaccine uptake and effectiveness (Technical Appendix 2).

We tested the model in 3 populations: persons in the United States as a whole; England and Wales; and Alaska Natives (defined as the indigenous residents of Alaska). These populations reflect major diversity in Hib epidemiology and vaccine policy (*1**,**3**,**4*). In the United States, Hib conjugate vaccines were first recommended in 1988 as a single dose for children 18 months of age, and in 1991, they were recommended as a primary series starting at 2 months of age, with a booster dose at 12–15 months. In England and Wales, Hib conjugate vaccines were introduced in 1992 as a primary series starting at 2 months of age and a 1-time catch-up campaign for children <4 years of age.

We began the simulations for the US and Alaska Native populations in 1980 and for the England and Wales population in 1985. We used census data to determine the size and age structure of each population. In the starting year, we divided the populations among the model states so that Hib transmission was in or nearly in equilibrium. Modeling and subsequent analyses were all implemented by using SAS version 9.2 (SAS Institute Inc., Cary NC, USA).

### Evaluating Model Fit

We verified model fit by using pseudo-R^2^ to compare the age-specific point prevalence of Hib colonization from the model with observed prevalence data in the time period before vaccine introduction (*4**,**12**–**14*). In a similar manner, we compared the annual incidence rate of invasive Hib among children <5 years of age from the simulated populations with observed incidence data (*1**,**3**,**4**,**15**–**23*).

### Effects of Vaccine Shortage in the United States

Before the 2007–2009 vaccine shortage, Merck & Co., Inc. (Whitehouse Station, NJ, USA) and Sanofi Pasteur (Bridgewater, NJ, USA) were licensed to produce Hib vaccines for the United States. The shortage was triggered when Merck recalled certain lots of their Hib vaccine and suspended vaccine production. In Merck’s Hib vaccine, the Hib polyribosylribitol phosphate (PRP) polysaccharide is conjugated to *Neisseria meningitidis* outer membrane protein (OMP). PRP-OMP conjugate vaccines induce a strong immune response with a first dose at 2 months of age and are given as a 2-dose primary series (*24*). In Sanofi Pasteur’s vaccine, PRP is conjugated to tetanus toxoid (T). For the primary series, PRP-T vaccines achieve antibody titers comparable to those achieved by PRP-OMP vaccines, but PRP-T vaccines require a 3-dose primary series (*24*). *Haemophilus* b conjugate (HbOC) vaccine, a third Hib vaccine formerly used in the United States, couples PRP oligosaccharides to CRM_197_ (cross-reacting material 197, a nontoxic mutant of diphtheria toxin). HbOC vaccines have immunogenic properties similar to those for PRP-T and require a 3-dose primary series (*24*).

For the US population, we modeled the effect of an extended vaccine shortage to explore what might have happened if the shortage had lasted >18 months (*10*). We first ran the model assuming that vaccine coverage from 2008 onward remained the same as that in 2007 (a complete series scenario). In this scenario, 50% of vaccinated children were assumed to receive PRP-OMP vaccine and 50% PRP-T vaccine, as determined by Merck and Sanofi Pasteur’s preshortage Hib vaccine market shares. We then ran the model assuming that the booster dose was deferred for all children starting in 2008 (a no-booster scenario) and that all vaccinated children received PRP-T. Last, we ran the model assuming that the booster dose was deferred starting in 2008 and that primary series coverage decreased by 10 percentage points, as suggested by some coverage surveys during the shortage (*25*) (a no-booster minus scenario). Again, all children were assumed to receive PRP-T. We compared annual incidence of invasive Hib in children <5 years of age under these 3 scenarios.

For the Alaska Native population we modeled the effect of switching Hib vaccines starting in 2010. During June 1991–1995 and from July 1997 onward, Alaska Native populations received PRP-OMP; during January 1996–June 1997, they received HbOC vaccines. Hib incidence in Alaska Native children rose in 1996–1997 when HbOC was used, prompting a switch back to PRP-OMP (*4*). During the 2007–2009 shortage, PRP-OMP vaccines from the Strategic National Stockpile were used for Alaska Natives (*9*). If Merck had not returned its vaccine to the market as expected, Alaska Natives would eventually have had to switch to PRP-T vaccines. To predict the effects of this switch, we compared predicted incidence in children <5 years of age from 2 models: 1 model assumed that PRP-OMP continued to be used from 2010 onward, and the other model assumed that PRP-T was used starting in 2010. We also modeled the effect of 1-time PRP-T booster campaigns, which occurred in conjunction with the switch to PRP-T, for all children 1–4 or 5–10 years of age.

### Alternative Approaches to Vaccine Introduction

This model can also be used to explore strategies for introducing Hib conjugate vaccines to new populations. To illustrate this strategy, we modeled hypothetical vaccination programs in 2 populations with the age distribution and transmission patterns of the United States or of the Alaska Native population.

We compared predicted Hib incidence in children <5 years of age in 4 vaccination scenarios in the hypothetical populations: 1) a primary series starting at 2 months of age and a booster dose at 12–15 months of age; 2) only a primary series starting at 2 months of age; 3) only a single dose at 12–15 months of age; and 4) a primary series at 2 months of age and a 1-time catch-up campaign for children <5 years of age. We assumed the strategies used PRP-T for all vaccine doses, with 90% vaccine coverage achieved within 3 years of vaccine implementation.

### Sensitivity Analyses

All model parameters taken from the literature are estimates based on samples of the population, and these estimates have some degree of uncertainty. We conducted detailed sensitivity analyses to determine whether our model conclusions would differ had we used different parameter values (Technical Appendix 3). We ran the model 10,000 times, each time randomly varying 3 parameters, and we looked for individual parameters and combinations of parameters that caused major differences between observed and modeled incidence in children <5 years of age. To test the effect of the rate of recovery from colonization, we also refit the model and ran several vaccination scenarios under extreme values for this parameter.

## Results

### Model Fit

**Figure 2:**
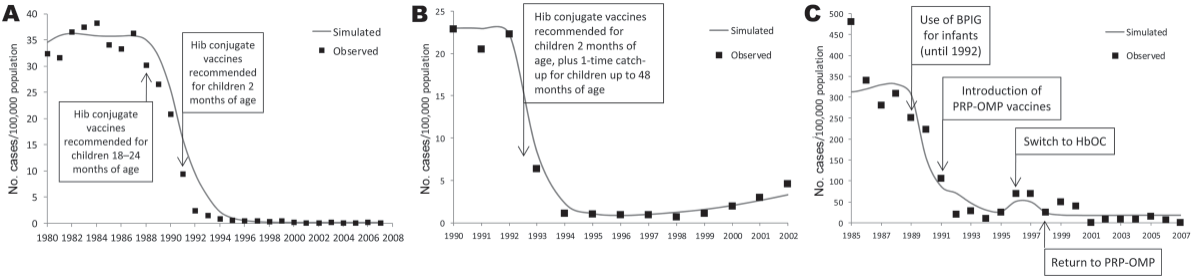
Observed and simulated incidence of invasive *Haemophilus influenzae* type b in children <5 years of age in 3 populations: (A) United States; (B) England and Wales; (C) Alaska Natives. PRP-OMP, Hib polyribosylribitol phosphate polysaccharide conjugated to *Neisseria meningitidis* outer membrane protein; HbOC, *Haemophilus* b conjugate vaccine; PRP-T, polyribosylribitol phosphate polysaccharide conjugated to tetanus toxoid.

The model accurately reproduced the observed prevalence of carriage by age group before vaccine introduction for the United States as a whole (pseudo-R^2^ 0.74) and for Alaska Natives (pseudo-R^2^ 0.98), the 2 populations for which carriage data were available. The model also accurately reproduced the observed annual incidence of invasive Hib in children <5 years of age in the United States (pseudo-R^2^ 0.97), in England and Wales (pseudo-R^2^ 0.91), and among Alaska Natives (pseudo-R^2^ 0.90) (Figure 2). Of note, the model captured the rise in Hib incidence in the United Kingdom beginning in 1999 and the rise in invasive disease among Alaska Natives that was associated with the switch to HbOC vaccines in 1996/1997.

### Force of Infection

For the United States and England and Wales, the best-fit force of infection suggests that Hib transmission before introduction of vaccine was driven by children 2–4 years of age (Table). Persons of all ages in both populations are primarily colonized through contact with children in this age group. For example, in the United States population, the annual force of infection on children <2 years of age was 36.3 infections/1,000 children, of which 24.3 (66.9%) were caused by children in the 2- to 4-year-old age group.

**Table:** Estimated annual prevaccination force of infection from Hib infectious persons to persons with susceptible, no-antibody status and estimated annual prevalence of Hib colonization in 3 modeled populations, stratified by age group*

Susceptible population, age group, y	Hib infections caused by infectious persons/1,000 susceptible persons, by age group, y				Total no. cases
	0–1	2–4	5–9	>10	
United States					
0–1	0.2	24.3	11.6	0.2	36.3
2–4	0.1	77.4	1.4	0.3	79.2
5–9	10.1	136.2	15.5	2.0	163.8
>10	7.0	78.5	9.2	0.8	95.5
Prevalence	1.1%	2.9%	5.3%	3.2%	NA
England and Wales					
0–1	0.6	15.4	10.7	1.7	28.4
2–4	3.1	62.8	11.2	1.9	79.0
5–9	10.1	133.6	16.8	1.9	162.5
>10	6.6	78.4	8.4	2.4	95.8
Prevalence	1.0%	3.0%	4.9%	3.0%	NA
Alaska Natives					
0–1	109.5	5.8	52.3	1.2	168.8
2–4	28.4	15.8	49.2	5.4	98.8
5–9	28.9	144.3	357.8	5.9	536.9
>10	28.4	21.7	80.3	64.7	195.1
Prevalence	5.2%	3.9%	9.9%	4.5%	NA

*Values are no. infections except as indicated. Data are based on Hib simulation model. Hib, *Haemophilus influenzae* type b; NA, not applicable.

Furthermore, the model suggests that the dynamics of Hib transmission are different in Alaska Native populations than in the other 2 modeled populations. In Alaska Native populations, most Hib transmission before introduction of vaccine came through contact with children 5–9 rather than 2–4 years of age (Table). A stronger element of assortative mixing was also present, in that children <2 years of age acquired infection from other children <2 years of age, and persons >10 years of age acquired infection from other persons >10 years of age.

### Model Predictions of Possible Effects of Hib Vaccine Shortage

**Figure 3:**
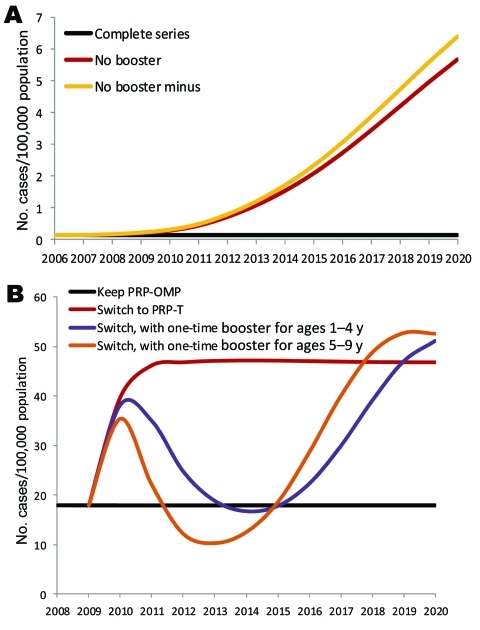
Predicted effects of extended *Haemophilus influenzae* type b (Hib) vaccine shortage on the incidence of invasive Hib disease in the United States (A) and of switching from PRP-OMP to PRP-T vaccine in the Alaska Native population (B). See text for complete description of shortage scenarios. PRP-OMP, Hib polyribosylribitol phosphate polysaccharide conjugated to *Neisseria meningitidis* outer membrane protein; PRP-T, polyribosylribitol phosphate polysaccharide conjugated to tetanus toxoid.

If the Hib vaccine shortage and deferral of the 12–15 month booster dose in the United States extended indefinitely, the model predicts relatively little change in the incidence of invasive Hib in children <5 of age for the first 3 years under either shortage scenario (Figure 3, panel A). Beginning in 2011, the model predicts that Hib incidence would increase more substantially in the no-booster shortage scenario (from 0.14 cases/100,000 children in 2007 to 0.72/100,000 in 2012 and 5.7/100,000 by 2020), with slightly greater increases in the no-booster minus shortage scenario.

If Alaska Native populations would have had to switch from PRP-OMP to PRP-T vaccine, the model predicts that the incidence of Hib in children <5 years of age would more than double (from 17.9 cases/100,000 children in 2009 to 46.2/100,000 in 2011) (Figure 3, panel B). Given that children 5–9 years of age appear to drive transmission in Alaska Native populations, we modeled the effect of adding a 1-time vaccination campaign for children 5–9 years of age in 2010 to the switch from PRP-OMP to PRP-T vaccine. This model predicts that such a vaccination campaign would keep the incidence of Hib below that for the PRP-T vaccine scenario for 8 years (Figure 3, panel B). The effect of a 1-time booster campaign for children 1–4 years of age was similar (Figure 3, panel B).

### Alternative Approaches to Vaccine Introduction

**Figure 4:**
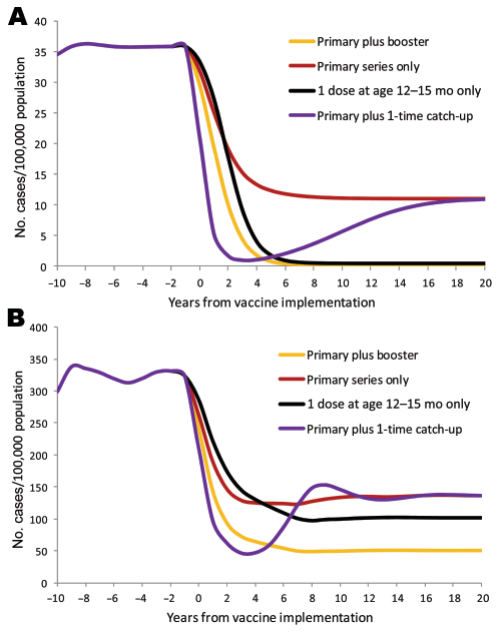
Predicted effects of different vaccination programs on the incidence of invasive *Haemophilus influenzae* type b disease, applied to a population with age structure and transmission dynamics like the United States population (A) and like the Alaska Native population (B).

In a hypothetical population with the age distribution and transmission patterns of the United States, the most effective strategy for vaccine introduction would have been to introduce the vaccine as a primary series plus a booster at 12–15 months of age (Figure 4, panel A). Using PRP-T vaccines, we found that this strategy resulted in a rapid decline in incidence and the lowest equilibrium incidence, i.e., 0.22 cases/100,000 children <5 years of age. A strategy of offering only 1 dose of vaccine at 12–15 months of age, without a primary series, was predicted to have nearly as great an effect on Hib incidence, with an equilibrium incidence of 0.47 cases/100,000 children <5 years of age. Strategies using a primary series only or a primary series with a 1-time catch-up campaign were much less effective, resulting in an equilibrium incidence of 11.0 cases/100,000 children <5 years of age.

In a hypothetical population with the age distribution and transmission patterns of Alaska Natives, the most effective strategy with PRP-T vaccines again would have been vaccinating with a primary series and a booster dose at 12–15 months, which yielded an equilibrium incidence of 50.4 cases/100,000 children <5 years of age (Figure 4, panel B). Both the primary series alone and the primary series with a 1-time catch-up yielded equilibrium incidence rates of 136.2 cases/100,000 children <5 years of age. As with the US population, the strategy of a single dose at 12–15 months of age was superior to a primary series alone, with or without a 1-time catch-up. However, the equilibrium incidence of 101.7 cases/100,000 children <5 years of age was substantially higher than that for the primary plus booster strategy.

### Sensitivity Analyses

We found that the model was robust to variations in all parameters except the mean rate of recovery from colonization (Technical Appendix 3). To see whether conclusions about Hib epidemiology from our model would differ on the basis of the value of this recovery rate, we tried fitting the model assuming a fast and slow recovery rate. The results showed that our conclusions about Hib dynamics and the effect of vaccination programs would be unchanged even under extremely different values for the mean rate of recovery from carriage.

## Discussion

We have developed a flexible model of Hib transmission and disease that can be applied to multiple contexts. This model can account for many essential features of Hib epidemiology, including the rapid decline in Hib incidence in the United States after vaccine introduction; the rise in Hib incidence in the United Kingdom 7 years after the catch-up campaign; and the increase in Hib incidence among Alaska Native populations when vaccine was switched from PRP-OMP to HbOC in 1996.

Our model suggests several essential insights into the epidemiology of Hib and into the design of Hib vaccination programs. First, our model suggests that in the United States and England and Wales, Hib transmission is driven by children 2–4 years of age. This is in contrast to prior Hib simulation models, which have suggested that transmission to persons of a given age group primarily occurs from others of the same age group (assortative mixing) (*26**–**28*). One model suggests that adults also play a major role across age groups (*27*). However, those transmission patterns do not explain the rapid decline in Hib incidence in the United States in 1988–1990, when Hib conjugate vaccine was only being offered to children 18–24 months of age. During that time, incidence declined even among children <1 year of age, an effect that is only possible if children >18 months of age are major drivers of Hib transmission.

Second, our model suggests that Hib transmission dynamics differ across populations. Unlike the findings from the best-fit model for the United States and England and Wales, the best-fit model for the Alaska Native population suggests that transmission is mainly driven by children 5–9 years of age, with some element of assortative mixing. These differences have major consequences for the design of Hib vaccination programs. For example, our model suggests that in the United States and England and Wales, giving 1 dose at 12–15 months of age would be nearly as effective, at a population level, as a full primary series plus a booster at 12–15 months of age. In contrast, offering only a single dose at 12–15 months would be considerably less effective for the Alaska Native population. Furthermore, PRP-T vaccine was predicted to be much less effective than PRP-OMP vaccine for Alaska Native populations because in Alaska Natives, the force of infection is high even for young infants and PRP-OMP stimulates protective antibodies after the first dose at 2 months of age. A high force of infection in Alaska Native infants is consistent with the observed jump in Hib incidence in Alaska Natives in 1996–1997 with the switch to HbOC vaccine, which does not induce protective antibodies until the third dose at 6 months of age (*11*). Planning an optimal vaccination program should include some assessment of the Hib transmission dynamics in the target population. Our model can be used to estimate those dynamics from the age-specific prevalence of colonization and age-specific incidence of invasive Hib.

The World Health Organization recommends that all routine infant vaccination programs include conjugate Hib vaccines in infancy, with or without a booster later in life (*29*). Most countries with Hib vaccination programs are in line with these guidelines (*30*). Our study suggests 2 potential practical applications for the design of Hib vaccination programs. First, there may be populations for which a policy of a single dose at 12–15 months of age would reduce the invasive Hib nearly as much as would a 3-dose primary series plus a booster. Furthermore, in some populations a single dose at 12–15 months may reduce Hib disease more than a 3-dose primary series without a booster. Additional exploration of the potential utility of a single dose of Hib conjugate vaccine at 12–15 months of age as a complete routine immunization schedule is needed. Second, countries planning to add Hib vaccines to their routine immunization programs could apply this model to local or regional data on Hib disease and colonization to characterize the potential effect of vaccination regimens under consideration.

Our study has a few limitations worth highlighting. First, as with all models, ours necessarily simplifies the underlying reality. We combined all persons >10 years of age into a single group because Hib colonization and incidence data were insufficient to reliably model more age groups within this broad category. The estimated transmission dynamics for this age group thus represent an average of adolescents and adults and may mask heterogeneity between these groups. Second, a model is only as good as the source data; if the estimates of model parameters from the literature are inaccurate, our model may be inaccurate. We conducted extensive sensitivity analyses to explore this (Technical Appendix 3), and found that the model is robust to variation in most parameters. The exception is the rate of recovery from Hib colonization. This finding makes sense because duration of infectiousness is a major determinant of disease transmission. However, we are reassured that this sensitivity does not affect our conclusions because using widely varied values for this rate does not change the basic model conclusions. Third, we assume that immunity following natural infection is the same as immunity following vaccination, while, in reality, natural infection may induce longer-lasting protection. Our model could reproduce Hib incidence in England and Wales more accurately than a model that used different parameters for vaccine-induced versus natural immunity (*28*). This suggests the difference in protection between vaccination and natural infection may not be epidemiologically essential; however, exploring this is a topic for future research.

A strength of our study is that our model is complex enough to successfully model Hib in a variety of populations yet simple enough that the transmission parameters can be estimated from relatively limited carriage and incidence data. A second strength is that, like Leino et al. (*27*), we used an iterative process to refine initial estimates of the transmission parameters. This process enables greater flexibility than that available when constraining the matrix to certain combinations of parameters (*26*) or choosing initial values by hand without further refinement (*28*).

Our Hib simulation model can be a useful tool for public health planners in countries that are considering implementing Hib vaccination programs and for countries that must respond to Hib vaccine shortages. The model suggests the importance of young children in the transmission of Hib, the need for a dose at 12–15 months of age to maintain herd immunity against Hib disease, and the importance of evaluating Hib transmission dynamics for optimizing vaccine programs.

## Supplementary Material

Technical Appendix 1 The following set of partial differential equations defines the rates at which the simulated population moves between model states.

Technical Appendix 2 We used published and unpublished data to set values for the model parameters.

Technical Appendix 3 As shown in Technical Appendix 2 Table 1 our Haemophilus influenzae type b (Hib) simulation model uses published research studies to define values many of the model parameters such as birth and death rates; protective effects of low and high antibody levels; and rates of recovery from colonization and disease.
