# Supplementary material for: Modeling Insights into Haemophilus influenzae Type b Disease, Transmission, and Vaccine Programs
Source: Emerg Infect Dis. 2012 Jan;18(1):13–20. doi: 10.3201/eid1801.110336 (PMC3310093; doi:10.3201/eid1801.110336)
Supplement: Technical Appendix 1 — The following set of partial differential equations defines the rates at which the simulated population moves between model states. [file 11-0336-Techapp1_2p.pdf]

Article DOI: http://dx.doi.org/10.3201/eid1801.110336

Modeling Insights into *Haemophilus influenzae* Type b Disease, Transmission, and Vaccine Programs

Technical Appendix 1

Model Structure

The following set of partial differential equations defines the rates at which the simulated population moves between model states:

$$\frac{\partial NS}{\partial t}+\frac{\partial NS}{\partial a}=\mu(t,a)X(t)+\omega_L LS(t,a)+\omega_{BPIG} I(t,a)-\left[\text{[illegible]}(t)+\lambda(t,a)+\gamma(t,a)\varepsilon(a)+\delta_{BPIG}(t,a)\right]NS(t,a)$$

$$\frac{\partial I}{\partial t}+\frac{\partial I}{\partial a}=\delta_{BPIG}(t,a)NS(t,a)-\omega_{BPIG} I(t,a)$$

$$\frac{\partial NC}{\partial t}+\frac{\partial NC}{\partial a}=\lambda(t,a)NS(t,a)-\left[\text{[illegible]}(t)+\rho_C+\sigma(a)+\gamma(t,a)\varepsilon(a)\right]NC(t,a)$$

$$\frac{\partial LS}{\partial t}+\frac{\partial LS}{\partial a}=\omega_H HS(t,a)-\left[\text{[illegible]}(t)+\omega_L+\lambda(t,a)(1-\alpha_L)+\gamma(t,a)\varepsilon(a)\right]LS(t,a)$$

$$\frac{\partial LC}{\partial t}+\frac{\partial LC}{\partial a}=\lambda(t,a)(1-\alpha_L)LS(t,a)-\left[\text{[illegible]}(t)+\rho_C+\sigma(a)(1-\beta_L)+\gamma(t,a)\varepsilon(a)\right]LC(t,a)$$

$$\frac{\partial HS}{\partial t}+\frac{\partial HS}{\partial a}=\rho_C\left[NC(t,a)+LC(t,a)+HC(t,a)\right]+\rho_D D(t,a)+\gamma(t,a)\varepsilon(a)\left[NS(t,a)+LS(t,a)\right]-\left[\nu(t)+\omega_H(a)+\lambda(t,a)(1-\alpha_H)\right]HS(t,a)$$

$$\frac{\partial HC}{\partial t}+\frac{\partial HC}{\partial a}=\lambda(t,a)(1-\alpha_H)HS(t,a)+\gamma(t,a)\varepsilon(a)[NC(t,a)\ +LC(t,a)]-\left[\nu(t)+\rho_C+\sigma(a)(1-\beta_H)\right]HC(t,a)$$

$$\frac{\partial D}{\partial t}+\frac{\partial D}{\partial a}=\sigma(a)\left[NC(t,a)+(1-\beta_L)LC(t,a)+(1-\beta_H)HC(t,a)\right]-\left[\nu(t)+\rho_D\right]D(t,a)$$

In which:

- NS, NC, LS, LC, HS, HC, D, and I are population states, where N=No antibody, L = Low antibody, H = High antibody, S = Susceptible, C = Colonized, D = Diseased, and I = Immune; X(t) is the total population.
- μ(t,a) and ν(t) are time-dependent birth and death rates, respectively. Birth rate also depends on age as individuals are only born into the age=0 group
- ω_L is the rate at which low antibody wanes to no antibody and $\omega_H(a)$ is the age-dependent rate at which high antibody wanes to low antibody
- λ(t,a) is the time- and age-dependent force of infection
- γ(t,a) is the time- and age-dependent rate of vaccination, and ε(a) is the age-dependent vaccine take rate
- σ(a) is the age-dependent rate of invasive disease among colonized persons
- α_L and α_H are the efficacy of low and high antibody at preventing colonization
- β_L and β_H are the efficacy of low and high antibody at preventing invasive disease
- ρ_C and ρ_D are the rates of recovery from colonization and invasive disease, respectively
- $\delta_{BPIG}(t,a)$ is the time- and age-dependent rate of BPIG use (for Alaska Native populations only), and ω_{BPIG} is the rate of waning of BPIG protection.
