# Supplementary material for: Modeling Insights into Haemophilus influenzae Type b Disease, Transmission, and Vaccine Programs
Source: Emerg Infect Dis. 2012 Jan;18(1):13–20. doi: 10.3201/eid1801.110336 (PMC3310093; doi:10.3201/eid1801.110336)
Supplement: Technical Appendix 2 — We used published and unpublished data to set values for the model parameters. [file 11-0336-Techapp2_8p.pdf]

Article DOI: http://dx.doi.org/10.3201/eid1801.110336

Modeling Insights into *Haemophilus influenzae* Type b Disease, Transmission, and Vaccine Programs

Technical Appendix 2

Model Parameters and Implementation

Model Parameters

We used published and unpublished data to set values for the model parameters (Technical Appendix 2 Table 1). Parameters assumed to be constant across populations include rates of recovery from colonization and disease, the proportion of vaccinations that induce high antibody levels (the vaccine "take rate"), the rate at which antibody levels wane, and the protective efficacy of low and high antibody levels against colonization and disease. We used studies of the duration of *Haemophilus influenzae* type b (Hib) colonization (*1–3*) and disease (*4*) to determine the rates of recovery from colonization and disease. We estimated the vaccine take rates based on antibody responses to Hib vaccination (*5*,*6*), assuming antibody titers of <0.15 μg/mL post-vaccination indicate lack of response. Rates of antibody waning were estimated from antibody titers at different time points post-vaccination and from prior Hib modeling studies (*5*,*7*). We estimated protection against colonization and disease associated with low and high antibody levels from studies of vaccine effectiveness (*8–10*).

The remaining model parameters were allowed to vary across populations. We estimated birth and death rates from vital statistics data (*11–15*). We used published and unpublished data on vaccine coverage by age and year to estimate rates of vaccination (*16–25*) (Centers for Disease Control and Prevention, unpub. data). To implement vaccination in the model, we assume that vaccination with the primary series takes effect at the completion of the primary series. That is, vaccination is treated as a single event that takes place at four months (PRP-OMP) or six months (PRP-T, HbOC) of age. We assume that single doses given after the first year of life take effect at the time of vaccination.

To parameterize the age- and time-specific force of infection ($\lambda(t,a)$) we first partitioned the population into four age classes: 0 to <2 years of age; 2 to <5 years of age, 5 to <10 years of age, and ≥10 years of age. The force of infection on susceptibles in age class *i* is then:

$$\lambda(t,i) = \sum_{j=1}^{4} c_{ij} * Y_j(t) * p_{ij}$$

where *j* represents the four age classes, c_{ij} is the rate at which susceptibles in age class *i* contact persons in age class *j*; Y_j is the proportion of persons in age class *j* who are infectious; and p_{ij} is the probability of transmission from *j* to *i* given contact between susceptible and infectious persons (*26*). The terms c_{ij} and p_{ij} can be combined into a single transmission coefficient, β_{ij}. The collection of β_{ij} values forms a Who Acquires Infection from Whom (WAIFW) matrix. To estimate the WAIFW matrix in the United States and Alaska Native populations we first tried a wide variety of possible matrices, varying the degree of assortative mixing and the relative importance of each age group; for each matrix we ran the model to equilibrium and determined which matrix gave the best fit between observed and predicted age-specific prevalence of colonization prior to the introduction of Hib vaccines (*22,27–29*). We then took the best-fit matrix and used maximum likelihood estimation (MLE) to refine the matrix parameters and get the best fit to the observed prevalence of colonization (Technical Appendix 2 Table 2).

Finally, to estimate the age-specific rate at which disease develops in colonized persons, we used data on the incidence of invasive Hib by age (*22,30*) and the duration of Hib disease to estimate the point prevalence of invasive Hib in each population. We then computed the age-specific ratio of Hib colonization prevalence to Hib disease prevalence. We fit a variety of functions to this ratio and chose the function with the best fit to the data based on the Akaike Information Criteria. We then use MLE to refine the parameter values for the best fitting function to best match the age-specific incidence of disease in each population.

For the England and Wales population, insufficient data are available on the prevalence of Hib colonization by age to estimate the WAIFW matrix and rate of disease among colonized persons. We assumed the rate of disease among colonized persons was the same as in the United

States population and adapted the United States WAIFW matrix to England and Wales age-specific Hib incidence (*31*) by using MLE.

To account for the use of bacterial polysaccharide immunoglobulin (BPIG) to passively immunize Alaska Native infants during July 1, 1989–April 30, 1992, we added an additional Immune model state. Newborns receiving BPIG (*23*) move to the Immune state and return to the No antibody, Susceptible state as BPIG wanes.

Implementation

We divided the population into n = 520 age groups, by week of age from birth to age 10 years. We treated persons ≥10 years of age as a single age group because little data exist on age-specific Hib colonization and incidence for persons ≥10 years of age. Within each age group, the set of partial differential equations that govern the model reduces to a set of ordinary differential equations. To run the model, we moved the population between the model states within each age group using the ordinary differential equations. We then incremented time by one week and aged the population by moving individuals from age n to age n + 1, with newborns entering the model at age n = 0.

Calculating the Force of Infection

After fitting the models, we calculated the age-specific force of infection in each population by running the models and determining the simulated age-specific point prevalence of Hib carriage just prior to vaccine implementation. We multiplied the WAIFW matrix by the age-specific prevalence of carriage to get the age-specific force of infection.

Technical Appendix 2 Table 1. Values and sources for parameters used in the model, by population*

Parameter name	Parameter symbol†	United States Value	United States Source(s)	England and Wales Value	England and Wales Source(s)	Alaska Natives Value	Alaska Natives Source(s)
Rate of recovery from colonization	ρ_C	2.1759/y	(*1–3*)	Same for all populations			
Rate of recovery from disease	ρ_D	33.2067/y	(*4*)	Same for all populations			
Proportion of vaccines inducing immunity	$\varepsilon(a)$						
Vaccine at <1 year of age		0.99	(*5*)	Same for all populations			
Vaccine at ≥1 year of age		1	(*6*)	Same for all populations			
Protection of high antibody against colonization	α_H	0.96	(*9*)	Same for all populations			
Protection of high antibody against disease	β_H	0.98	(*8*)	Same for all populations			
Protection of low antibody against colonization	α_L	0	(*10*)	Same for all populations			
Protection of low antibody against disease	β_L	0.9	(*8*)	Same for all populations			
Rate of waning from high to low antibody	$\omega_H(a)$		(*5,7*)				
<1 year of age		4.9987/y		Same for all populations			
1–1.99 years of age		0.3287/y		Same for all populations			
≥2 years of age		0.1983/y		Same for all populations			
Rate of waning from low to no antibody	ω_L	0.0939/y	(*5,7*)	Same for all populations			
Force of infection from outside population‡	part of $\lambda(t,a)$	0.0005/y	(*32*)	Same for all populations			
Birth rate per 1,000 population	$\mu(t,a)$	15.236/y	(*14*)	12.40/y	(*12,13*)	Varies over time	(*14*)
Death rate per 1,000 population	$\nu(t,a)$	8.713/y	(*11*)	10.64/y	(*12,13*)	Varies over time	(*11,15*)
Rate of disease among colonized: $x + ye^{-z \times \text{age}}$	$\sigma(a)$						
x		2.25×10^{-6}	MLE	2.25×10^{-6}	MLE	3.50×10^{-5}	MLE
y		0.002614	MLE	0.002614	MLE	0.01591	MLE
z		1.0446/y	MLE	0.02002/wk	MLE	0.0371/wk	MLE
Rate of Hib vaccination	$\gamma(t,a)$	Varies over time	(*16–19*)	Varies over time	(*20,21*)	Varies over time	(*22,25*)
Rate of BPIG use in newborns	$\delta_{BPIG}(t,a)$	Not used		Not used		Varies over time	(*23*)
Rate of waning of BPIG	ω_{BPIG}	Not used		Not used		Varies by age and time§	(*23*)

*MLE, maximum-likelihood estimation; Hib, *Haemophilus influenzae* type b; BPIG, bacterial polysaccharide immunoglobulin.
†Corresponds to symbols used in Technical Appendix 1 (wwwnc.cdc.gov/EID/pdfs/11-0336-Techapp1.pdf).
‡Force of infection from within the population is found in Table 2 of the article.
§Time-varying because the number of doses of bacterial polysaccharide immunoglobulin varied over time.

Technical Appendix 2 Table 2. Final Who Acquires Infection from Whom matrices for the United States; England and Wales; and Alaska Native populations*

United States population

		Age group of infectious persons (j), y			
		0–1	2–4	5–9	≥10
Age of susceptible persons (i), y	0–1	0.02	0.83	0.22	0.01
	2–4	0.01	2.66	0.03	0.01
	5–9	0.96	4.68	0.29	0.06
	≥10	0.66	2.70	0.17	0.03

England and Wales population

		Age group of infectious persons (j), y			
		0–1	2–4	5–9	≥10
Age of susceptible persons (i), y	0–1	0.05	0.51	0.22	0.06
	2–4	0.29	2.08	0.23	0.06
	5–9	0.97	4.43	0.34	0.06
	≥10	0.63	2.60	0.17	0.08

Alaska Native population

		Age group of infectious persons (j), y			
		0–1	2–4	5–9	≥10
Age of susceptible persons (i), y	0–1	2.11	0.15	0.53	0.03
	2–4	0.55	0.40	0.50	0.12
	5–9	0.56	3.68	3.61	0.13
	≥10	0.55	0.55	0.81	1.43

*Matrix values are the product of (the annual rate at which persons of age group i encounter persons of age group j) and (the probability of transmission given contact between susceptible in age group i and infectious in age group j).

References

1. Glode MP, Daum RS, Boies EG, Ballard TL, Murray M, Granoff DM. Effect of rifampin chemoprophylaxis on carriage eradication and new acquisition of *Haemophilus influenzae* type b in contacts. Pediatrics. 1985;76:537–42. PubMed

2. Glode MP, Schiffer MS, Robbins JB, Khan W, Battle CU, Armenta E. An outbreak of *Hemophilus influenzae* type b meningitis in an enclosed hospital population. J Pediatr. 1976;88:36–40. PubMed doi:10.1016/S0022-3476(76)80723-8

3. Michaels RH, Norden CW. Pharyngeal colonization with *Haemophilus influenzae* type b: a longitudinal study of families with a child with meningitis or epiglottitis due to H. influenzae type b. J Infect Dis. 1977;136:222–8. PubMed doi:10.1093/infdis/136.2.222

4. McConnell A, Tan B, Scheifele D, Halperin S, Vaudry W, Law B, et al. Invasive infections caused by *Haemophilus influenzae* serotypes in twelve Canadian IMPACT centers, 1996–2001. Pediatr Infect Dis J. 2007;26:1025–31. PubMed doi:10.1097/INF.0b013e31812f4f5b

5. Heath PT, Booy R, Azzopardi HJ, Slack MP, Bowen-Morris J, Griffiths H, et al. Antibody concentration and clinical protection after Hib conjugate vaccination in the United Kingdom. JAMA. 2000;284:2334–40. PubMed doi:10.1001/jama.284.18.2334

6. Käyhty H, Eskola J, Peltola H, Stout MG, Samuelson JS, Gordon LK. Immunogenicity in infants of a vaccine composed of *Haemophilus influenzae* type b capsular polysaccharide mixed with DPT or conjugated to diphtheria toxoid. J Infect Dis. 1987;155:100–6. PubMed doi:10.1093/infdis/155.1.100

7. McVernon J, Ramsay ME, McLean AR. Understanding the impact of Hib conjugate vaccine on transmission, immunity and disease in the United Kingdom. Epidemiol Infect. 2008;136:800–12. PubMed doi:10.1017/S0950268807009168

8. Eskola J, Käyhty H, Takala AK, Peltola H, Ronnberg PR, Kela E, et al. A randomized, prospective field trial of a conjugate vaccine in the protection of infants and young children against invasive *Haemophilus influenzae* type b disease. N Engl J Med. 1990;323:1381–7. PubMed doi:10.1056/NEJM199011153232004

9. Takala AK, Eskola J, Leinonen M, Käyhty H, Nissinen A, Pekkanen E, et al. Reduction of oropharyngeal carriage of *Haemophilus influenzae* type b (Hib) in children immunized with an Hib conjugate vaccine. J Infect Dis. 1991;164:982–6. PubMed doi:10.1093/infdis/164.5.982

10. Takala AK, Santosham M, Almeido-Hill J, Wolff M, Newcomer W, Reid R, et al. Vaccination with *Haemophilus influenzae* type b meningococcal protein conjugate vaccine reduces oropharyngeal carriage of *Haemophilus influenzae* type b among American Indian children. Pediatr Infect Dis J. 1993;12:593–9. PubMed doi:10.1097/00006454-199307000-00010

11. National Center for Health Statistics. National Vital Statistics System. HIST290: death rates for selected causes by 10-year age groups, race, and sex: death registration states, 1900–32, and United States, 1933–98 [cited 2010 Jun 14]. http://www.cdc.gov/nchs/nvss/mortality/hist290.htm

12. UK Office for National Statistics. Tables. Population Trends. Winter 1997;90:49–75 [cited 2010 Jun 18]. http://www.ons.gov.uk/ons/rel/population-trends-rd/population-trends/no--90--winter-1997/population-trends.pdf

13. UK Office for National Statistics. Tables. Population Trends. Winter 2002;110:42–70 [cited 2010 Jun 18]. http://www.ons.gov.uk/ons/rel/population-trends-rd/population-trends/no--110--winter-2002/population-trends.pdf

14. National Center for Health Statistics. Vital Statistics of the United States. 2003, vol I. Natality. 2005 [cited 2010 Jun 18]. http://www.cdc.gov/nchs/products/vsus.htm#natab2003

15. National Center for Health Statistics. Health, United States, 2009: with special feature on medical technology. 2010 [cited 2010 Jun 18]. http://www.cdc.gov/nchs/data/hus/hus09.pdf

16. Centers for Disease Control and Prevention. Vaccination coverage of 2-year-old children–United States, 1993. MMWR Morb Mortal Wkly Rep. 1994;43:705–9. PubMed

17. Centers for Disease Control and Prevention. State and national vaccination coverage levels among children aged 19–35 months–United States, April–December 1994. MMWR Morb Mortal Wkly Rep. 1995;44:613, 619, 621–3. PubMed

18. Centers for Disease Control and Prevention. Status report on the Childhood Immunization Initiative: national, state, and urban area vaccination coverage levels among children aged 19–35 months–United States, 1996. MMWR Morb Mortal Wkly Rep. 1997;46:657–64. PubMed

19. Centers for Disease Control and Prevention. National, state, and urban area vaccination coverage among children aged 19–35 months–United States, 2003. MMWR Morb Mortal Wkly Rep. 2004;53:658–61. PubMed

20. Trotter CL, Ramsay ME, Slack MP. Rising incidence of *Haemophilus influenzae* type b disease in England and Wales indicates a need for a second catch-up vaccination campaign. Commun Dis Public Health. 2003;6:55–8. PubMed

21. Annual COVER report: 2004/05: Summary of trends in vaccination coverage in the UK 1st April 1995 to 31st March 2005. 2005 [cited 2010 Jun 18]. http://www.hpa.org.uk/webc/HPAwebFile/HPAweb_C/1194947372171

22. Singleton R, Hammitt L, Hennessy T, Bulkow L, DeByle C, Parkinson A, et al. The Alaska *Haemophilus influenzae* type b experience: lessons in controlling a vaccine-preventable disease. Pediatrics. 2006;118:e421–9. PubMed doi:10.1542/peds.2006-0287

23. Singleton RJ, Davidson NM, Desmet IJ, Berner JE, Wainwright RB, Bulkow LR, et al. Decline of *Haemophilus influenzae* type b disease in a region of high risk: impact of passive and active immunization. Pediatr Infect Dis J. 1994;13:362–7. PubMed doi:10.1097/00006454-199405000-00006

24. Ward J, Brenneman G, Letson GW, Heyward WL. Limited efficacy of a *Haemophilus influenzae* type b conjugate vaccine in Alaska Native infants. The Alaska *H. influenzae* Vaccine Study Group. N Engl J Med. 1990;323:1393–401. PubMed doi:10.1056/NEJM199011153232006

25. Singleton R, Bulkow LR, Levine OS, Butler JC, Hennessy TW, Parkinson A. Experience with the prevention of invasive *Haemophilus influenzae* type b disease by vaccination in Alaska: the impact of persistent oropharyngeal carriage. J Pediatr. 2000;137:313–20. PubMed doi:10.1067/mpd.2000.107843

26. Anderson RM, May RM. Age-related changes in the rate of disease transmission: implications for the design of vaccination programmes. J Hyg (Lond). 1985;94:365–436. PubMed doi:10.1017/S002217240006160X

27. Hampton CM, Barenkamp SJ, Granoff DM. Comparison of outer membrane protein subtypes of *Haemophilus influenzae* type b isolates from healthy children in the general population and from diseased patients. J Clin Microbiol. 1983;18:596–600. PubMed

28. Michaels RH, Poziviak CS, Stonebraker FE, Norden CW. Factors affecting pharyngeal *Haemophilus influenzae* type b colonization rates in children. J Clin Microbiol. 1976;4:413–7. PubMed

29. Stillman EG. Occurrence of H. influenzae in throats of adults. Yale J Biol Med. 1945;18:37–40. PubMed

30. Wenger JD, Hightower AW, Facklam RR, Gaventa S, Broome CV. Bacterial meningitis in the United States, 1986: report of a multistate surveillance study. The Bacterial Meningitis Study Group. J Infect Dis. 1990;162:1316–23. PubMed doi:10.1093/infdis/162.6.1316

31. Ramsay ME, McVernon J, Andrews NJ, Heath PT, Slack MP. Estimating *Haemophilus influenzae* type b vaccine effectiveness in England and Wales by use of the screening method. J Infect Dis. 2003;188:481–5. PubMed doi:10.1086/376997

32. Coen PG, Heath PT, Barbour ML, Garnett GP. Mathematical models of *Haemophilus influenzae* type b. Epidemiol Infect. 1998;120:281–95. PubMed doi:10.1017/S0950268898008784
