# Supplementary material for: Modeling Insights into Haemophilus influenzae Type b Disease, Transmission, and Vaccine Programs
Source: Emerg Infect Dis. 2012 Jan;18(1):13–20. doi: 10.3201/eid1801.110336 (PMC3310093; doi:10.3201/eid1801.110336)
Supplement: Technical Appendix 3 — As shown in Technical Appendix 2 Table 1 our Haemophilus influenzae type b (Hib) simulation model uses published research studies to define values many of the model parameters such as birth and death rates; protective effects of low and high antibody levels; and rates of recovery from colonization and disease. [file 11-0336-Techapp3_6p.pdf]

Article DOI: http://dx.doi.org/10.3201/eid1801.110336

Modeling Insights into *Haemophilus influenzae* Type b Disease, Transmission, and Vaccine Programs

Technical Appendix 3

Sensitivity Analyses

Rationale

As shown in Technical Appendix 2 Table 1 (wwwnc.cdc.gov/EID/pdfs/11-0336-Techapp2.pdf), our *Haemophilus influenzae* type b (Hib) simulation model uses published research studies to define values many of the model parameters such as birth and death rates; protective effects of low and high antibody levels; and rates of recovery from colonization and disease. The true population values of these parameters are unknown; we merely have point and interval estimates of these parameters based on samples from the population. For example, the vaccine take rate parameter (the proportion of vaccinations that induce protective immunity) in persons ≥1 year of age comes from Käyhty et al. (reference *6* in Technical Appendix 2), where all 34 persons ≥1 year of age achieved protective antibody levels after vaccination with Hib conjugate vaccine. Thus, our modeled estimate for the vaccine take rate in persons ≥1 year of age is 1.0. However, with a sample size of 35 persons, a vaccine take rate as low as 0.9 would not be inconsistent with these data.

For modeling purposes, we must select a single value for each parameter, and in each case we choose the best estimate from the available data (typically the mean). However, the true parameter value in the population may be different from the value we choose for the model. To properly interpret the model results, it is essential to assess how sensitive the model is to the specific parameters values we chose. Returning to the above example of the vaccine take rate, we want to know whether our conclusions from the model would be different if we had used a take rate of 0.9 rather than 1.0. In this Technical Appendix we present detailed sensitivity analyses of our Hib simulation model. The goal of these analyses is to determine whether the conclusions of

our model depend on the specific parameter values used, or whether the model is robust to the uncertainty in the estimates of these parameters. This includes robustness to the specific values for individual parameters and for combinations of parameters.

Approach

The model uses 15 parameters that have been defined based on published studies. The model also includes parameters for the force of infection and the incidence of disease among the colonized, which were estimated as part of the model fitting process (Technical Appendix 2). We restricted our sensitivity analyses to the parameters based on the literature. Inference about the force of infection is part of the purpose of the model, and varying the values of the model output would not inform us about the sensitivity of the model to the other parameters.

For each of 15 parameters based on published studies, we define the point estimate and its SE from the published studies. The point estimates were used in the primary analysis as described in the main manuscript. Here, we make use of the SEs to explore how sensitive the model is to each of the 15 parameters and to combinations of the parameters.

For the sensitivity analyses, we ran 10,000 iterations of the simulation model on the United States population. In each iteration, we randomly selected 3 parameters to vary. We randomly sampled a value for each of those three parameters from a distribution defined by the parameter's point estimate and SE. We then ran the United States model from 1980 through 2000 using the sampled values of the three parameters and the point estimates for all remaining model parameters. We determined the predicted incidence of invasive Hib in children <5 years of age in 1987 (the last year before vaccination was started) and in 2000 for each iteration of the model.

For each of the 15 parameters, we then calculated the mean and SD of the mean for the modeled incidence in 1987 and 2000 across all iterations of the model where that parameter was allowed to vary. For any individual parameter, a large SD indicates that the model is sensitive to the value of that parameter. In contrast, a small SD indicates that the model is robust to the uncertainty in the estimation of that parameter.

The model may also be sensitive to certain combinations of parameters without being highly sensitive to the individual parameters. To explore this possibility, we looked at all two-way and three-way combinations of parameters, again calculating the SD of the mean incidence in 1987 and 2000 for all iterations where those parameters were varied together.

Results of Sensitivity Analyses

The observed annual incidence of invasive Hib per 100,000 children <5 years of age was 36.3 in 1987 and 0.23 in 2000. Across iterations, the mean modeled incidence matches the observed incidence in these years closely (Technical Appendix 3 Table 1). However, at first glance, it appears that the model is sensitive to the particular values of all the parameters. The SD of the predicted annual incidence per 100,000 in 1987 was ≥8.4 for all parameters. For example, when death rate was allowed to vary along with any other 2 parameters, the modeled incidence in 1987 had a mean of 36.1 and an SD of 8.7, which is a high degree of variability.

However, closer examination of the data shows that the variability is predominantly due to a single parameter: the rate of recovery from colonization (ρ_C). The SD for incidence in 1987 across iterations where ρ_C was varied is 23.8, which is an extreme amount of variability. The high SD in estimated incidence from varying the other parameters was largely due to iterations where ρ_C was varied along with the other parameters. When the variability of the remaining parameters was examined only among iterations where ρ_C was fixed (set to the mean), the SD was much smaller, never larger than 1.6 for incidence in 1987. Thus, we conclude that the model is highly sensitive to the value of ρ_C, and highly robust to the remaining individual parameters.

We further examined all 2-way and 3-way combinations of the parameters, excluding iterations where ρ_C was also varied. The SE for estimated incidence in 1987 was never >2.3 for any pairs or triads of parameters, and never >0.03 for incidence in 2000. This finding indicates that there were not pairs or triads of parameters to which the model is highly sensitive.

Additional Analyses

Because the model is highly sensitive to the rate of recovery from colonization, we further explored whether changes in this parameter would impact our conclusions from the model. We chose two extreme values for ρ_C — a fast recovery rate 2 SE higher than the mean value and a slow recovery rate 2 SD lower than the mean recovery rate. The mean (SE) duration of colonization from the literature was 0.46 years (168 days), which corresponds to ρ_C of 0.0417 recoveries per week among the colonized (references *1–3* in Technical Appendix 2). Two SE above this was a recovery rate of 0.0547 recoveries per week, corresponding to an average

duration of colonization of 128 days. Two SE below was a recovery rate of 0.0336 recoveries per week, an average duration of 208 days.

For each of the extreme values of ρ_C, we refit the “who acquires infection from whom” (WAIFW) matrix for the United States population. We compared the WAIFW matrices generated from the mean, extreme low, and extreme high rates of ρ_C to see whether our conclusions about the relative role of each age group for Hib transmission differs depending on the modeled value of ρ_C. In addition, we tested whether our conclusions about the impact of different vaccination strategies would differ based on the modeled rate of ρ_C. For this, we ran the United States model under three scenarios: using a primary series only, using a primary series and a booster, and using a single dose at 12–15 months only.

We found that the specific value used for ρ_C does not affect the conclusions we draw based on our model. Across all 3 values of ρ_C, our model suggests that children 2–4 years of age are the key drivers of Hib transmission in the United States (Technical Appendix 3 Table 2). Furthermore, across all 3 values of ρ_C our model suggests that using a single Hib dose in the second year of life would reduce Hib incidence more than a primary series in infancy with no booster dose (Technical Appendix 3 Figure).

Summary

Although the specific values of the model parameters must be defined from estimates of these values that are measured with uncertainty, this uncertainty does not impact our model’s conclusions. Other than the rate of recovery from colonization, any reasonable values for the model parameters alone or in combination can be substituted into the model without impacting the model output’s fit to observed incidence data. The model can be fit using a wide range of values for the rate of recovery from colonization and still result in similar conclusions about the epidemiology of Hib and the impact of Hib vaccination programs.

Technical Appendix 3 Table 1. Mean and SD of predicted annual incidence per 100,000 persons in 1987 and 2000 from the Hib simulation model, where the value of the listed parameter was randomly sampled from its distribution*****

	Incidence among all iterations where the parameter was sampled				Incidence excluding iterations where recovery from colonization was varied			
	1987		2000		1987		2000	
Parameter	Mean	SD	Mean	SD	Mean	SD	Mean	SD
Birth rate	35.8	8.4	0.16	0.04	35.6	0.9	0.15	0.01
Death rate	36.1	8.7	0.16	0.03	35.7	0.8	0.15	0.01

	Incidence among all iterations where the parameter was sampled				Incidence excluding iterations where recovery from colonization was varied			
	1987		2000		1987		2000	
Parameter	Mean	SD	Mean	SD	Mean	SD	Mean	SD
Vaccine take rate								
Age <1 year	35.9	8.8	0.16	0.03	35.6	0.8	0.15	0.01
Age ≥1 year	36.3	8.6	0.17	0.04	35.7	0.8	0.16	0.02
Outside force of infection	36.5	9.7	0.16	0.03	35.7	0.8	0.15	0.01
Rate of recovery from colonization	39.5	23.8	0.18	0.09	NA	NA	NA	NA
Rate of recovery from disease	36.1	9.5	0.16	0.03	35.7	0.7	0.15	0.01
Rate of waning from low to no antibody	36.5	9.6	0.16	0.04	35.6	0.8	0.15	0.01
Rate of waning from high to low antibody								
Age <1 year	36.3	8.8	0.16	0.04	35.7	0.7	0.15	0.01
Age 1 year	36.2	9.3	0.16	0.04	35.7	0.8	0.15	0.01
Age ≥2 years	36.5	9.5	0.16	0.03	35.7	0.8	0.15	0.01
Protection of low antibodies against colonization	35.1	9.4	0.14	0.03	34.7	1.6	0.14	0.02
Protection of low antibodies against disease	36.2	9.2	0.16	0.03	35.7	0.8	0.16	0.02
Protection of high antibodies against colonization	36.4	8.4	0.16	0.03	35.8	0.9	0.15	0.01
Protection of high antibodies against disease	36.3	9.1	0.16	0.04	35.7	0.8	0.15	0.01

*Hib, *Haemophilus influenzae* type b; NA, not applicable.

Table 2. Estimated "who acquires infection from whom" matrix in the United States population using three values for the rate of recovery from colonization*

Mean recovery rate (0.0417 recoveries per week)

		Age group of infectious persons (j), y			
		0–1	2–4	5–9	≥10
Age group of susceptible persons (i), y	0–1	0.02	0.83	0.22	0.01
	2–4	0.01	2.66	0.03	0.01
	5–9	0.96	4.68	0.29	0.06
	≥10	0.66	2.70	0.17	0.03

Extremely low recovery rate (0.0336 recoveries per week)

		Age group of infectious persons (j), y			
		0–1	2–4	5–9	≥10
Age group of susceptible persons (i), y	0–1	0.76	0.19	0.30	0.01
	2–4	0.01	2.15	0.02	0.01
	5–9	0.22	3.34	0.38	0.08
	≥10	0.12	1.94	0.23	0.02

Extremely high recovery rate (0.0547 recoveries per week)

		Age group of infectious persons (j), y			
		0–1	2–4	5–9	≥10
Age group of susceptible persons (i), y	0–1	0.22	1.03	0.27	0.01
	2–4	0.55	3.33	0.03	0.02
	5–9	1.76	5.59	1.46	0.08
	≥10	1.93	31.10	0.74	0.03

*Matrix values are the product of (the annual rate at which persons of age group i encounter persons of age group j) and (the probability of transmission given contact between susceptible in age group i and infectious in age group j)

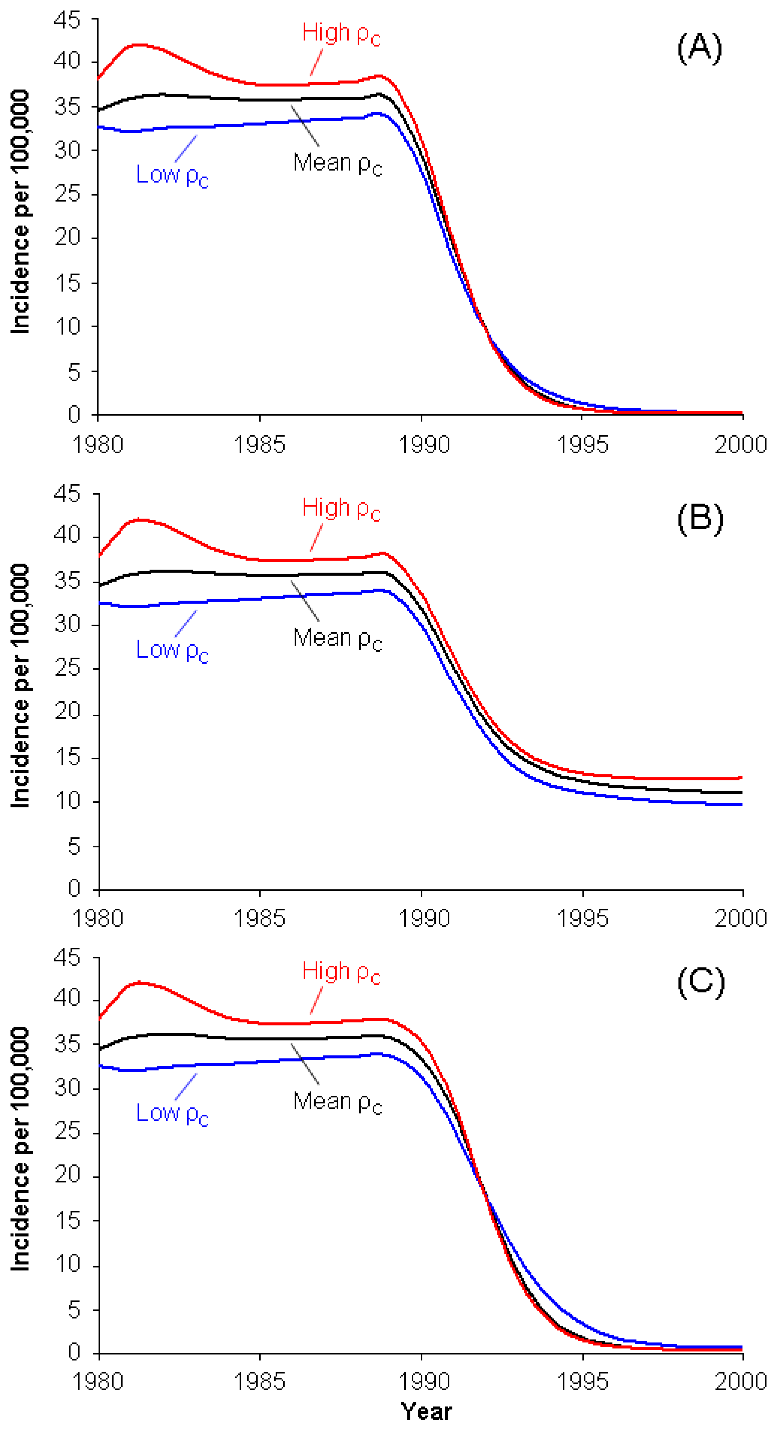

Technical Appendix 3 Figure. Predicted effects of different Hib vaccination strategies on incidence of Hib in children <5 years of age, in a United States-like population, under different assumptions about the rate of recovery from colonization (ρ_C). (A) Vaccination with primary series and booster dose; (B) Vaccination with primary series only; (C) Vaccination with a single dose at 12–15 months of age only.
